# Consistent FFP2-masking as part of reducing viral respiratory infections on medical wards for allogeneic hematopoietic stem cell transplantation

**DOI:** 10.1038/s41598-024-72646-y

**Published:** 2024-09-14

**Authors:** T. Richardson, D. Schütte, K. Feyer, L. Grass, M. Hallek, C. Scheid, F. Simon, T. Braun, M. Fürstenau, P. Gödel, U. Holtick

**Affiliations:** University of Cologne Hospital, Cologne, Germany

**Keywords:** Disease prevention, Haematological cancer

## Abstract

Patients undergoing allogenic hematopoietic stem cell transplantation (allo-HSCT) are highly susceptible to infections. The consequent use of masks on wards for allo-HSCT has been controversial in the past decades and was not common before the COVID-19 pandemic. We retrospectively compared incidence and outcomes of viral respiratory infections during allo-HSCT on our specialized ward between 01/2018 and 09/2020 to the era of FFP2 masking between 10/2020 and 10/2022 covering similar seasons of the year. Each group consisted of 150 matched patients. The usage of FFP2 masks reduced the incidence of viral respiratory infections from 20,7% to 2,0% (p < 0.001). This reduced the time on ward from a median of 26 days to 23.5 days (p = 0.002). It also resulted in less use of CT-scans (p = 0.003) and bronchoalveolar lavage procedures (p = 0.057). Median time to proof of infection was 21 days after admission in both groups. No difference was detected in progression free survival, hospital survival or non-relapse mortality (p = 0.78). Our retrospective results indicate that FFP2 masks worn by patients and hospital staff may help to significantly reduce the incidence of viral respiratory infections, including COVID-19, shorten the in-hospital time, and reduce costs without affecting survival.

## Introduction

Allogeneic hematopoietic stem cell transplantation (allo-HSCT) is a curative treatment modality for patients with hematologic malignancies and other conditions going along with a deeply impaired immune system which renders the patient susceptible to infections for several month. The use of masks on medical wards has been recommended to reduce the spread of infectious agents but was not common before the COVID-19 pandemic. With a lack of immunity against COVID-19, almost all countries made mask wearing mandatory for the general population and especially in hospitals^1^.

Our goal was to assess whether masking during COVID-19 reduced the overall incidence of respiratory infections with potential benefit to patient outcomes compared to pre-masking times on a ward for allo-HSCT.

Viral respiratory infections (RVIs) are among the most common infections after allo-HSCT. In recipients, RVIs occur in 8–22% in the first 100 days after transplantation with high differences between countries, wards, transplant strategies and outpatient-concepts^2^. The common pathogens for RVIs include the influenza virus, the parainfluenza virus (PIV), the respiratory syncytial virus (RSV), the human metapneumovirus (HMPV), the human coronavirus (HCoV) and the human rhinovirus (HRV). RVIs result in an increased morbidity in stem cell transplant recipients, with reported mortality rates of 6–50%, depending on the pathogen^3^.

Before the COVID-19 pandemic, wearing a medical face mask was mandatory for healthcare professionals when treating cases with detectedm respiratory symptoms as part of infections preventive measures. Despite increasing knowledge, specific data on in-hospital transmissions of respiratory viruses remain scarce. Lindsey et al. published data on transmissions in UK-hospitals between patients and staff and showed a reduction of staff-to-staff transmissions through masking and other control measures, whilst still not making half of the infections detected^4^. Partridge et al. published data on masking with surgical masks on a ward for allo-HSCT and showed a reduction of RVI’s from 19.7 to 7.3% per 100 patients whilst also showing a high tolerability^3^. Similar data had been shown by Sung et al. with a decrease of 16.9–8.3% in the setting of allo-HSCT when masking with surgical masks was implemented^5^.

The key difference between stem cell transplant patients and the general population is that the frequency and severity of complications following viral infections increase dramatically^6,7^, in particular, in case of a progression to an infection of the lower respiratory tract which often evolves to a life-threatening scenario^8^.

Before the pandemic, in case of respiratory symptoms isolation has been mandatory and early PCR-based testing using nasal/throat swabs was used. In case of a positive results masking was applied in contact. As part of the SARS-CoV-2 pandemic, further and stricter measures to prevent infection have been taken in healthcare facilities. Generalized testing of all patients, visitors and employees was introduced at the University Hospital of Cologne and other medical facilities during the pandemic. This was carried out at regular intervals. In addition, patients were tested for respiratory pathogens upon admission. These measures have shown to contain the spread of COVID-19, but also that of other seasonal respiratory viral infections^9^.

FFP2/N95 masks, a type of respiratory protective equipment that can filter out at least 94% of airborne particles, showed superior effects compared to surgical masks on the general spread of infection^10^.

They also protect against transmission of viruses when infected people wear a mask^11^. Our analysis aims to analyze whether wearing FFP2 masks in the inpatient area of a stem cell transplant has a general positive effect on the viral respiratory infections of recipients of an allogeneic bone marrow transplantation.

## Methods

We compared the incidence and outcomes of viral respiratory infections in patients receiving allo-HSCT on our specialized ward between 01/01/2018 and 09/30/2020 for the pre-COVID era, and 10/01/2020–10/31/2022 to the era of FFP2 masking.

We included 300 consecutive patients 18 years and older that received their transplantation at our institution. Each patient was only included once and only the admission for initial transplantation was counted, not any rehospitalizations. Clinical follow-up was performed as standard of care. Data collection and analysis ended at our pre-specified analysis completion date. All methods were carried out in accordance with relevant guidelines and regulations. It was conducted according to the Declaration of Helsinki and approved by the institutional review board of university of cologne. This project was approved by the Institutional ethics board of the University of Cologne registered under 23–182-retro. All patients had an informed consent to the use of their anonymous data when admitted for transplantation.

Each group consisted of 150 patients matched for different malignant entities, age, sex, used induction regimen, usage of ATG, stem cell source, Hematopoietic cell transplantation comorbidity index (HCT-CI), infection before transplant, donor choice and remission before transplant. To avoid selection bias, we used the last 150 patients before masking and the first 150 patients after masking.

Our ward consists of 14 single-bed rooms with anterooms and an additional ventilation. We have restricted entry doors to the ward and a complete change of clothes before accessing. Each room has a positive pressure system to prevent infection through air particles from outside. Additionally, windows cannot be opened. Patients remain on the ward until engraftment of neutrophils after transplant. Visitor rights are limited to one asymptomatic visitor per day and patient and included obligatory testing of COVID-19 during the pandemic. Surgical masks were recommended before the pandemic but not obligatory. During the pandemic they had to be worn. Staff and visitors with respiratory symptoms were not allowed on the ward.

Screening for respiratory viruses via nasal/throat-swab was performed through multiplex PCR assays and all patients with positive PCR results were considered. Before the pandemic no asymptomatic screening for respiratory infections was used before admission. With the begin of the pandemic infections were ruled out by obligatory testing before admission of symptomatic and asymptomatic patients as well as staff. Testing happened three days before and on the day of admission. During the pandemic staff and patients were tested for COVID-19 two time the week while always wearing FFP2-masks on the ward. Breaks for eating were done separately and in distance. All patients received an obligatory baseline low-dose chest CT at day of admission. In case of a positive PCR the transplantation was postponed, if possible.

**Fig. 1:**
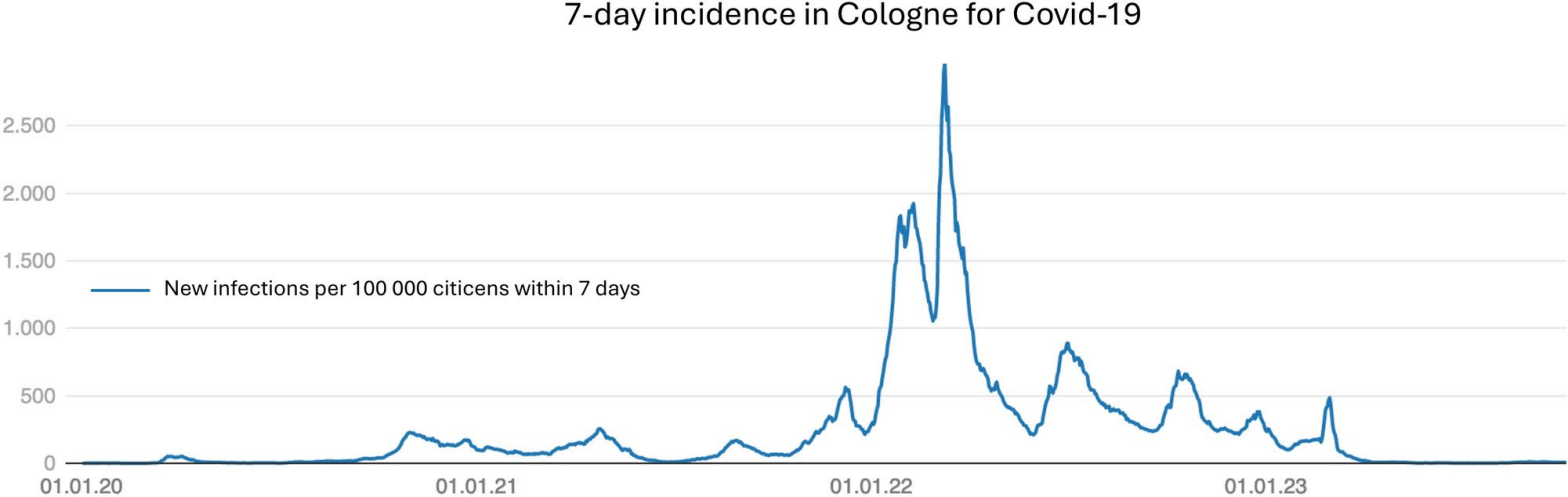
Showing 7-day incidence of COVID-19 in Cologne in the considered time frame. Graphic adapted from City of Cologne^12^.

To assess the effectiveness of wearing FFP2 masks on viral respiratory infections during allo-HSCT at the bone marrow transplant ward of the University Hospital Cologne, data from two different time periods were retrospectively considered. These two periods (01/01/2018–09/30/2020 and 10/01/2020–10/31/2022) differ in that there was no general masking mandate on the ward in the first period, surgical masks were only recommended for visitors. The second period begins on the day that the wearing of FFP2 masks at the University Hospital Cologne was made mandatory for staff, patients, and visitors due to the SARS-Cov-2 pandemic. In both periods, the data of 150 patients were compared. The first period consists of 33 months, while the second period only includes 25 months, since we were performing more transplants in the second term due to the availability of two more beds for most of the time. Both time periods include two high-incidence seasons for respiratory infections. Like in other countries the incidence of COVID-19 varied distinctive during the selected period. Figure 1 shows these fluctuations.

To collect specific data on the occurrence of an infection, it was recorded whether the patients already had a respiratory infection or a fungal infection of the lungs on admission or whether a respiratory infection occurred during their inpatient stay. We did not count patients with a respiratory viral infection (RVI) that pre-existed at admission towards the set of patients who acquired their infection during the stay, even if the RVI was repeatedly detected.

For the statistical analysis, the R Language for Statistical Computing, Version 4.1.3 was used. Proportions were compared using a Chi-squared test, whereas continuous variables were assessed for differences using the non-parametric Mann–Whitney U test due to a lack of normality. Proportions were compared using a Chi-squared test. Continuous variables were assessed for differences using the non-parametric Mann-Whitney U test due to a lack of normality.

## Results

We retrospectively analyzed 300 consecutive patients treated on our ward. Overall, the proportion of male patients was 61.3% and the proportion of female patients was 38.7%. The median age was 55 years. Median HCT-CI was one. 58% of patients were treated for acute leukemias the rest for a variety of mostly malignant hematological diseases. Detailed patient characteristics are described in Table 1.

**Table 1:** Patient characteristics.

	**Before masking mandate (N = 150)**	**After masking mandate (N = 150)**	**Overall (N = 300)**
Age			
Mean (SD)	51.1 (13.9)	52.2 (16.2)	51.6 (15.1)
Median [Min, Max]	54.0 [20.0, 75.0]	56.0 [17.0, 78.0]	55.0 [17.0, 78.0]
Sex			
Male	86 (57.3%)	98 (65.3%)	184 (61.3%)
Female	64 (42.7%)	52 (34.7%)	116 (38.7%)
Diagnosis			
ALL	17 (11.3%)	18 (12.0%)	35 (11.7%)
AML	73 (48.7%)	66 (44.0%)	139 (46.3%)
B-NHL (incl. CLL)	12 (8.0%)	14 (9.3%)	26 (8.7%)
Benign Hematological Disorders	2 (1.3%)	4 (2.7%)	6 (2.0%)
Hodgkin’s Lymphoma	2 (1.3%)	5 (3.3%)	7 (2.3%)
Multiple Myeloma	6 (4.0%)	3 (2.0%)	9 (3.0%)
Other	3 (2.0%)	3 (2.0%)	6 (2.0%)
Other Myeloid Neoplasias	29 (19.3%)	28 (18.7%)	57 (19.0%)
T-NHL	6 (4.0%)	9 (6.0%)	15 (5.0%)
Duration of stay post transplant			
Mean (SD)	32.3 (19.4)	26.7 (12.2)	29.6 (16.6)
Median [Min, Max]	26.0 [4.00, 138]	23.5 [2.00, 87.0]	25.0 [2.00, 138]
Missing	1 (0.7%)	10 (6.7%)	11 (3.7%)

Patients in our analysis had a median time of 156 days between first diagnosis and transplantation. In over 95% of cases, peripheral stem cells were used as stem cell source. Conditioning regiments used were reduced intensity conditioning (RIC) in 64% and 61% and myeloablative (MAC) in 36% and 39% of patients (pre- and post-masking, respectively). Donors were mostly matched and unrelated, with a proportion of 22% and 24% matched related donors in each group. Rabbit anti-thymocyte-globulin (ATG) was used in 42.7% of patients in both groups, respectively. In each group, almost one third of patients received post-transplant cyclophosphamide. Further systemic immunosuppression regimen was mostly calcineurin inhibitors in combination with mycophenolate mofetil (MMF), with equal numbers in both time periods.

**Fig. 2:**
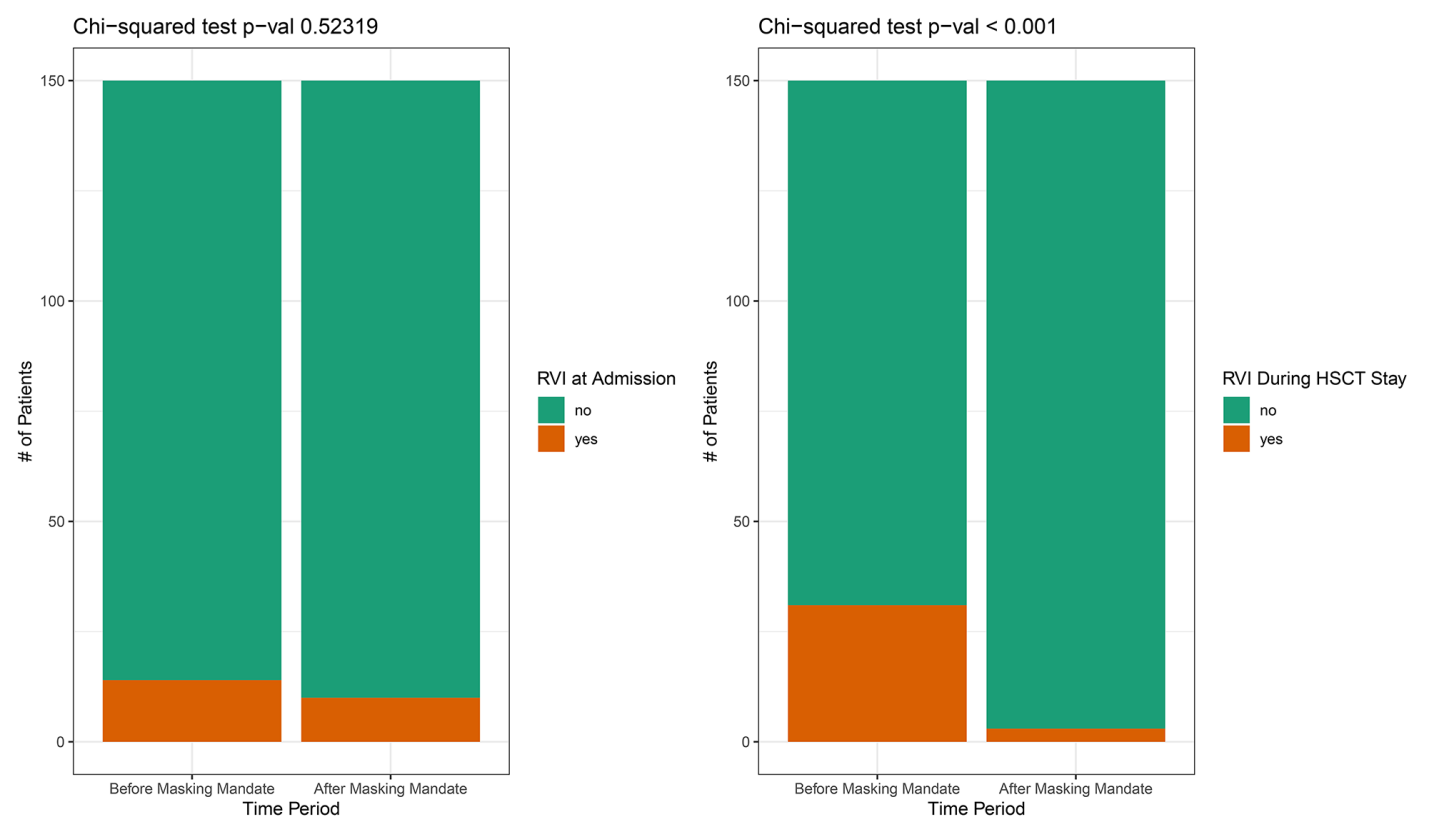
Comparison of incidence of viral respiratory infections.

After correcting for infections that were diagnosed at admission, we found 31 RVIs prior to and just 3 RVIs after masking became mandatory. Accordingly, measures taken during the pandemic, including the usage of FFP2 masks, reduced the incidence of viral respiratory infections during allo-HSCT from 20,7% (31/150) to 2,0% (3/150) compared to pre-masking times with significantly different distributions in the two groups (Mann-Whitney U p < 0.001, Fig. 2). Proven pathogens in the era before obligatory masking were 19 cases of rhinovirus, 6 of parainfluenza, 4 of coronavirus (not covid-19), 2 of metapneumovirus and 1 bocavirus with one patient having 2 RVIs during his stay. After masking was implemented, just 3 cases of rhinovirus were diagnosed.

**Fig. 3:**
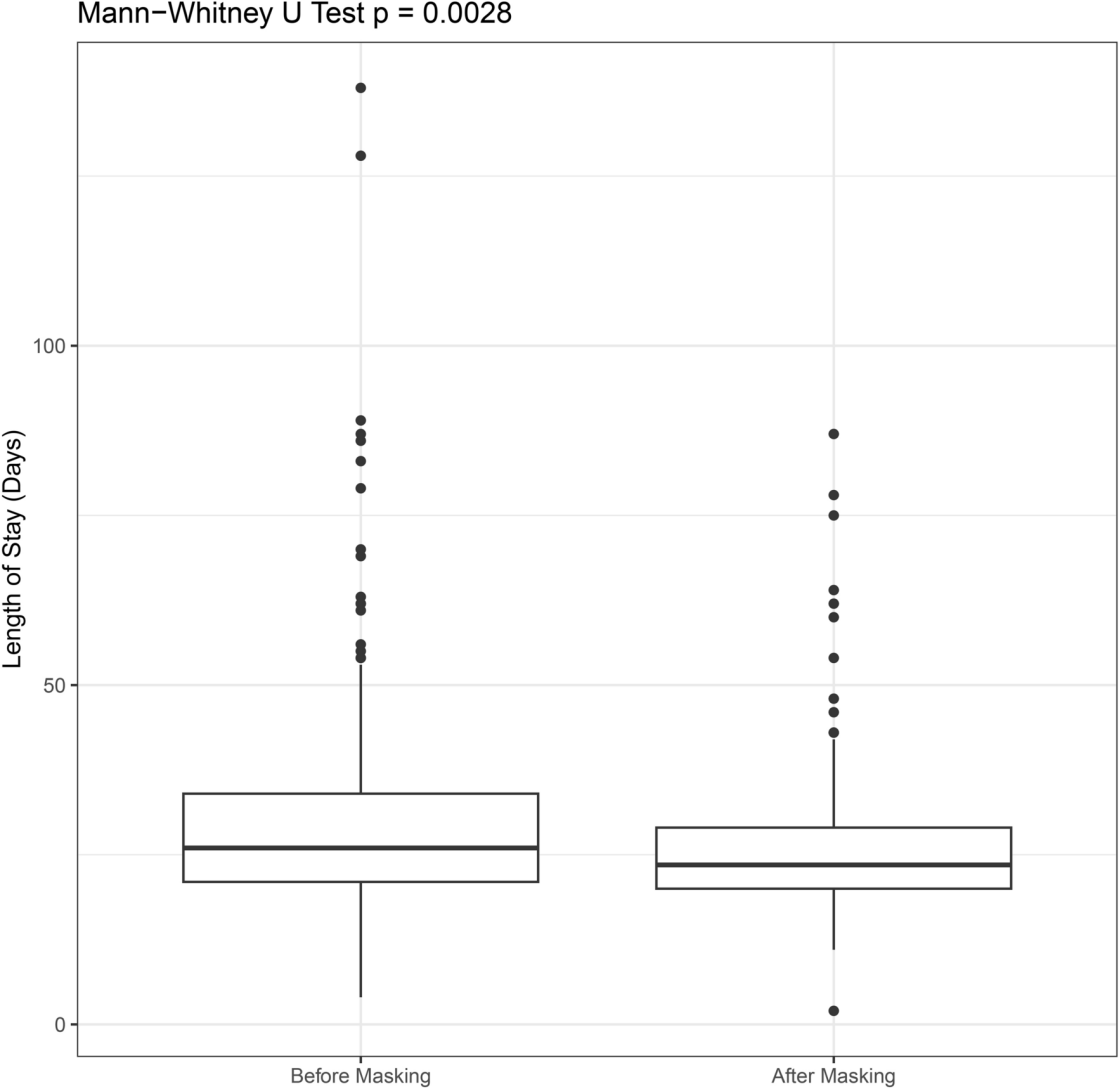
Comparison of length of in-hospital stay.

This reduction in respiratory infections was associated with a significant reduction of the time on ward from a median of 26 days to 23.5 days (p < 0.005) (Fig. 3).

A further decrease was found in the number of chest-CTs and BALs performed. In the first time period, 32 additional CTs and 14 BAL procedures were performed for diagnostic measures. In the 150 patients during the masking period, the number was lower with 13 additional CTs (p = 0.003) and 5 BALs (p = 0.057) performed.

The first and second group of patients had a median follow up of 32.3 and 9.6 months respectively. Median time to proof of the infection occurred 21 days after admission in both groups. No difference was found in 30-day overall survival, progression free survival or in hospital survival, (chi-square, p-value = 0.78). Both groups showed the same number of bacterial infections proven in blood cultures. Two cases of cytomegalovirus pneumonia were diagnosed in the first 150 patients. Fungal infections of the lungs were treated in 20 patients in the first and 15 patients in the second group, respectively.

In addition, there was no significant difference in viral infections already detected on admission, with 14 infections in the first group and 10 infections in the second group (Fig. 2).

## Discussion

Our retrospective analysis shows that respiratory infections, including COVID-19, were significantly reduced on a ward for allo-HSCT during the pandemic. We observed a significant drop in the incidence of infections from 20.7% to 2.0%.. These effects cannot be contributed to a single measure partaken in the fight against COVID-19. Increased awareness of spreading infections, measures of hygiene and social distancing all contributed to reduced contamination. A separate consideration of these measures is difficult. But even before the pandemic tolerance for working with symptoms of infection were low on the transplant ward and hygiene was taken seriously with these vulnerable patients. Puerta et al have shown that wearing protective masks has a positive influence on the spread and transmission of viral respiratory pathogens in this immunosuppressed collective with data focusing on the outpatient setting^9^. Even though there was an asymptomatic testing of patients and staff during the second era, which was not performed before COVID-19, the incidences remained low, underlining the effect of the measure implemented during the epidemic. This is underlined by the fact that during COVID-19 all patients and staff was for infection twice weekly via PCR, making the sample per patient ratio a lot higher. This may be attributed to the efficacy of masking in reducing transmission of viral pathogens in an inpatient setting^13^, since masking was the biggest single change carried out during the pandemic. Wards for allogeneic transplantation are well suited for comparing infection reduction measures because they also attach great importance to them outside of a pandemic.

The protective effect of masks both on the wearer and on people in the immediate vicinity through the effect of filtration of the breathing and ambient air has been shown multiple times^8,11,14^. Therefore, the alleviating effect we observed on the infection process in the context of allo-HSCT adds evidence to this data. Since surgical masks were not obligatory without symptoms before the pandemic our results show the asymptomatic transmission that may be prevented.

The median length of the in-hospital stay was significantly reduced from 26 days to 23.5 days, hereby potentially decreasing risks of nosocomial infections^15^, lowering costs and increasing patients’ quality of life whilst also extending the number of patients that can be treated due to higher capacities.

Lower numbers of respiratory infections lead to lower numbers of CT scans and BAL procedures performed whilst not translating into increased overall survival or reduced need for intensive care monitoring. Nevertheless, reducing unnecessary procedures lowered radiation exposure and risks associated with BALs like peri-procedural bleeding. Furthermore, these examination methods may be associated with relevant costs for the hospital and patients^16^. Not having an increased non-relapse mortality in our vulnerable patients during a pandemic remains a success by itself.

After allo-HSCT, pulmonary GvHD is reported in 3–15% of patients, often triggered by respiratory infections. Reducing the incidence of viral infections can potentially contribute to prevent this hard-to-treat complication of allo-HSCT^17^.

On the other hand, studies evaluating the downsides of wearing face masks have been published depicting concerns about masking during a physically demanding work on the wards as well as concerns about difficulties in communication, especially non-verbal, and emotional detachment from the patients^18^. Wearing masks frequently over a long period of time can lead to an increase in headaches, drowsiness, and a feeling of shortness of breath and exertion^19^. Additionally, some patients experienced a reduced sense of autonomy through the mandatory measure of wearing a mask without giving the affected person a choice^20^.

Our patients were able to take off their masks when alone in their room and most patients appreciated the extra effort for their safety through masking.

**Fig. 4:**
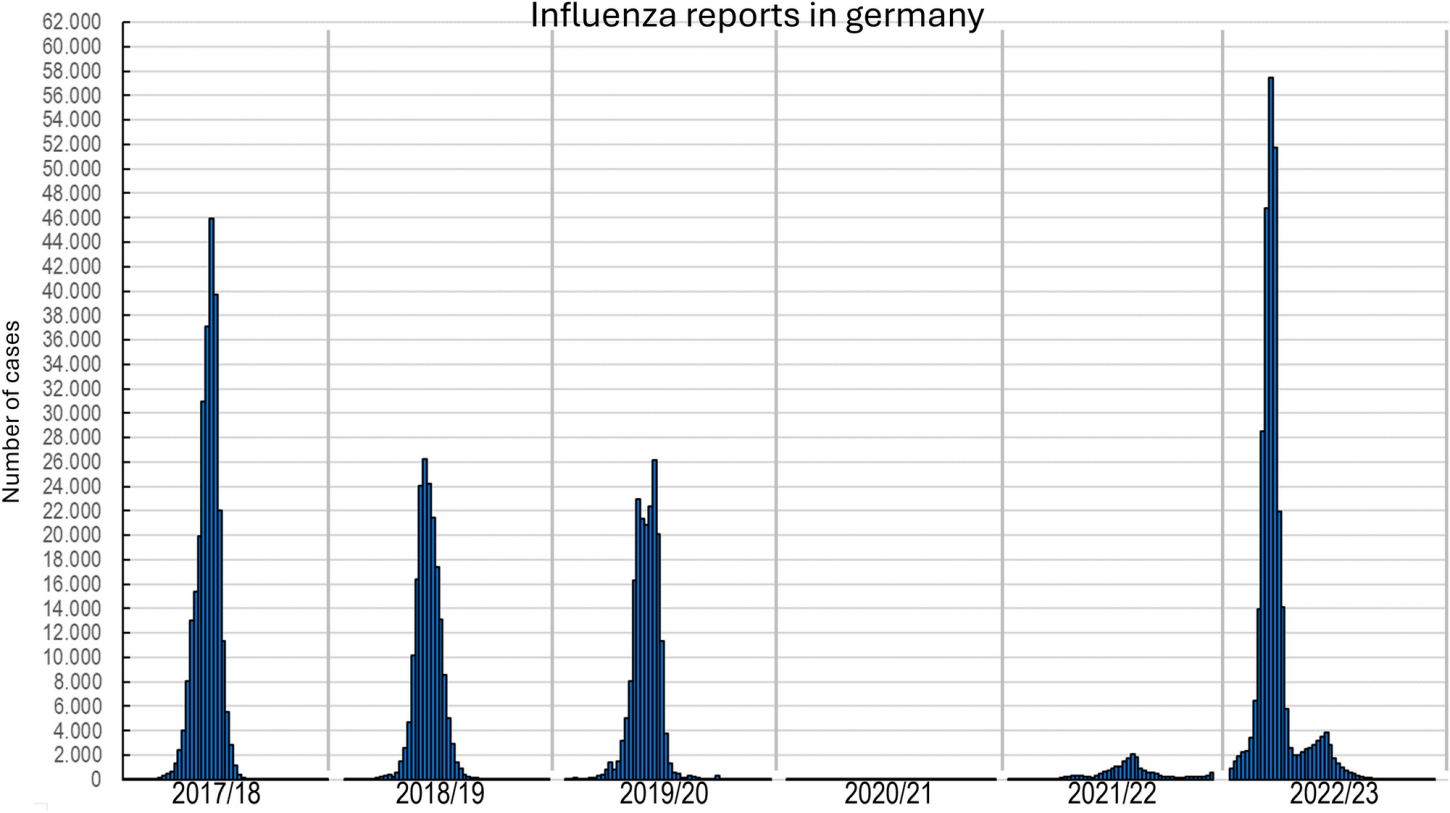
Overview of weekly laboratory-confirmed influenza reports since the 2017/18 season. The seasonal sections shown correspond to the reports from week 40 to week 39 of the following year. Graphic adapted from Robert-koch-institution Germany^12^.

In addition to the methodological limitations of the retrospective approach of our analysis, we lack multi-variate statistics to detect the possible effect of a general reduction in population-level respiratory infections during the COVID-19 pandemic into account. Reduction of influenza during the pandemic is shown in Fig. 4.

The changes imposed on society, hospital staff and patients during the pandemic were diverse and far-reaching. On the other hand, the frequency of infections at admission to our ward was comparable between the two time periods underlining the susceptibility of our patients and showing only a mild reduction in the era of masking. At note, during COVID-19 screening at admission was performed making the comparison difficult, detecting possible asymptomatic patients that may have remained undetected before screening.

Another limitation is the fact that other measures of reducing transmissions were implemented during the pandemic, such as increased testing for respiratory infections, intensified hygiene measures and a generally elevated awareness of the risk of contagion. These variables beyond masking might explain a part of the observed reduction in RVIs. Additionally, apart from COVID-19, other respiratory infections were less common on a population-level in the second period due to general measures of containing transmissions of respiratory infections. Whether this effect could also be described in immunosuppressed patients is unclear.

To our knowledge this is the most extensive analysis of the effects of FFP2 masking on a ward for allo-HSCT. We were able to show a significant reduction in the transmission of RVI’s. We thus hypothesize that FFP2-masks aid to not only reduce respiratory infections, irradiation exposure and procedural side-effects. It might also lower the overall cost of allogeneic stem cell transplantation and provide a safer environment for our highly vulnerable patients. In the future, prospective studies need to be conducted to test this hypothesis.

## Data Availability

All data for this project can be provided upon request.
