# Author Correction: Consistent FFP2-masking as part of reducing viral respiratory infections on medical wards for allogeneic hematopoietic stem cell transplantation

**DOI:** 10.1038/s41598-025-87738-6

**Published:** 2025-02-19

**Authors:** T. Richardson, D. Schütte, K. Feyer, L. Grass, M. Hallek, C. Scheid, F. Simon, T. Braun, M. Fürstenau, P. Gödel, U. Holtick

**Affiliations:** University of Cologne Hospital, Cologne, Germany

Correction to: *Scientific Reports* 10.1038/s41598-024-72646-y, published online 14 September 2024. 

The original version of this Article contained some errors, where the difference between respiratory viral and overall respiratory infections prior to transplantation as well during the transplant stay within the research group were incorrectly communicated.

In the Abstract,

“The usage of FFP2 masks reduced the incidence of viral respiratory infections from 22.1 to 2.1% (p < 0.005).”

now reads:

“The usage of FFP2 masks reduced the incidence of viral respiratory infections from 20,7% to 2,0% (p < 0.001).”

In the Methods section,

“To collect specific data on the occurrence of an infection, it was recorded whether the patients already had a respiratory infection or a fungal infection of the lungs on admission or whether a respiratory infection occurred during their inpatient stay.”

“For the statistical analysis, the R Language for Statistical Computing, Version 4.1.3 was used.”

now reads:

“To collect specific data on the occurrence of an infection, it was recorded whether the patients already had a respiratory infection or a fungal infection of the lungs on admission or whether a respiratory infection occurred during their inpatient stay. We did not count patients with a respiratory viral infection (RVI) that pre-existed at admission towards the set of patients who acquired their infection during the stay, even if the RVI was repeatedly detected.”

“For the statistical analysis, the R Language for Statistical Computing, Version 4.1.3 was used. Proportions were compared using a Chi-squared test. Continuous variables were assessed for differences using the non-parametric Mann-Whitney U test due to a lack of normality.”

In the Results,

“Measures taken during the pandemic, including the usage of FFP2 masks, reduced the incidence of viral respiratory infections during allo-HSCT significantly from 22.1 to 2.0% compared to pre-masking times (p < 0.005, Fig. 2). Proven pathogens in the era before obligatory masking were 19 cases of rhinovirus, 6 of parainfluenza, 4 of coronavirus (not COVID-19), 2 of metapneumovirus and 1 bocavirus. After masking was implemented, just 3 cases of rhinovirus were diagnosed.”

**Fig. 2:**
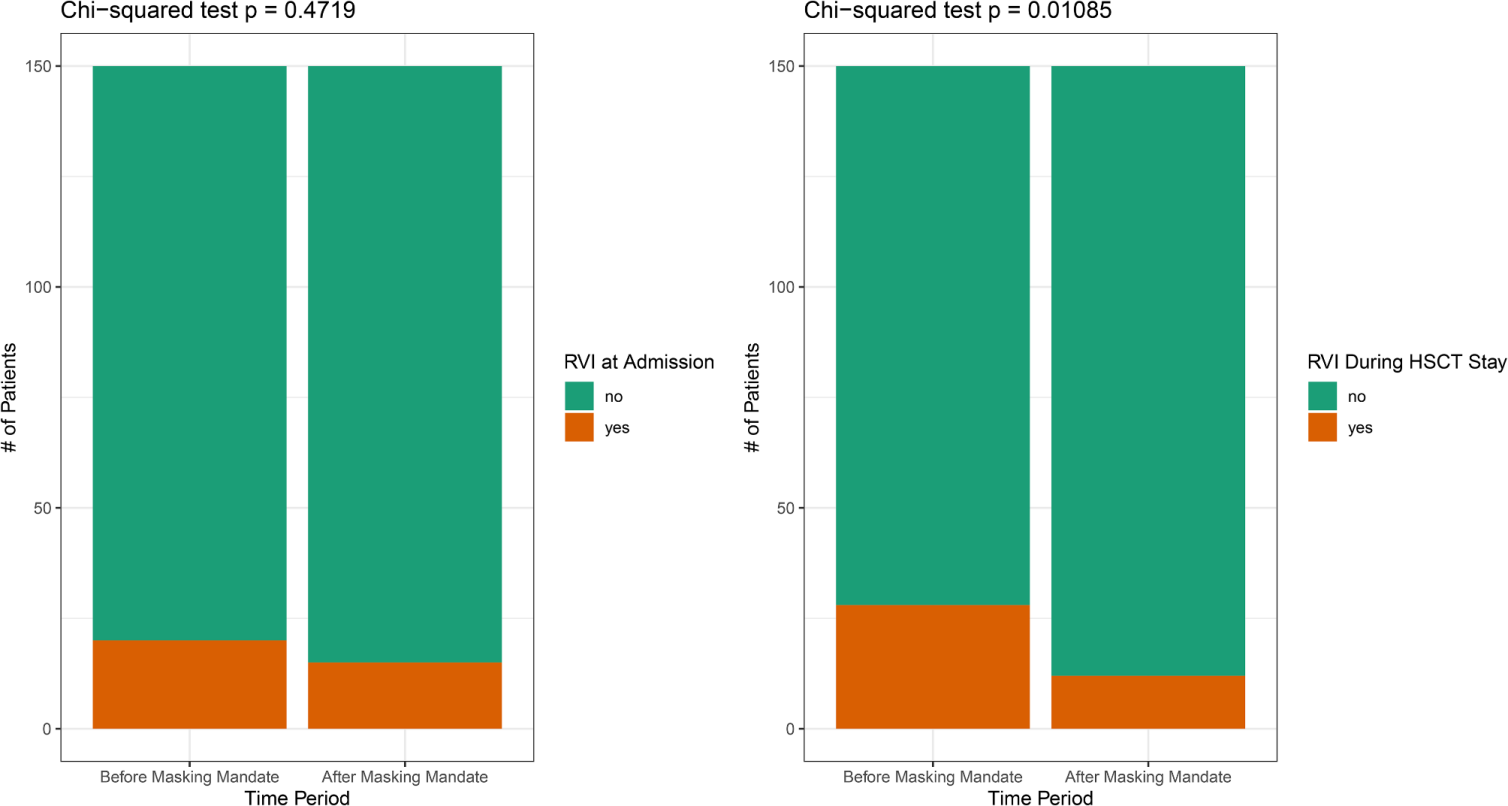
Comparison of incidence of viral respiratory infections.

“In addition, there was no significant difference in viral infections already detected on admission, with 20 infections in the first group and 15 infections in the second group (Fig. 1).”

now reads:

“After correcting for infections that were diagnosed at admission, we found 31 RVIs prior to and just 3 RVIs after masking became mandatory. Accordingly, measures taken during the pandemic, including the usage of FFP2 masks, reduced the incidence of viral respiratory infections during allo-HSCT from 20,7% (31/150) to 2,0% (3/150) compared to pre-masking times with significantly different distributions in the two groups (Mann-Whitney U p < 0.001, Figure 2). Proven pathogens in the era before obligatory masking were 19 cases of rhinovirus, 6 of parainfluenza, 4 of coronavirus (not covid-19), 2 of metapneumovirus and 1 bocavirus with one patient having 2 RVIs during his stay. After masking was implemented, just 3 cases of rhinovirus were diagnosed.”

“In addition, there was no significant difference in viral infections already detected upon admission, with 14 infections in the first group and 10 infections in the second group (Fig. 2).”

In addition, Figure 2 was incorrect. The original Figure 2 and accompanying legend appear below.

In the Discussion,

“We observed a significant drop in the incidence of infections from 22.1 to 2.1%.”

now reads:

“We observed a significant drop in the incidence of infections from 20.7% to 2.0%.”

The original Article has been corrected.

